# Editorial: Heat Acclimation for Special Populations

**DOI:** 10.3389/fphys.2020.00895

**Published:** 2020-07-29

**Authors:** Andrew T. Garrett, Neil S. Maxwell, Julien D. Périard, Caroline Sunderland

**Affiliations:** ^1^Department of Sport, Health and Exercise Science, University of Hull, Hull, United Kingdom; ^2^School of Sport and Service Management, University of Brighton, Brighton, United Kingdom; ^3^University of Canberra Research Institute for Sport and Exercise (UCRISE), Canberra, ACT, Australia; ^4^School of Science and Technology, Nottingham Trent University, Nottingham, United Kingdom

**Keywords:** exercise, heat stress, heat training, heat acclimatization, heat therapy

This heat acclimation for special population's Research Topic questions the “one size fits all” approach for heat adaptation and that it may not be appropriate for all populations. Therefore, to highlight these differences we endeavored to collect a set of studies on how heat acclimation may benefit a wide range of special populations who have specific needs.

We have published 12 articles in this Research Topic and defined four main areas of research. (a) an epidemiological approach and the aging process; (b) understanding physiological mechanisms and a novel heat acclimation method; (c) adaptation to the heat for special populations including males, females, military personnel and Paralympic athletes; and (d) the use of heat therapy for special populations. We have summarized the most noteworthy evidence of each study in these research areas.

## An Epidemiological Approach and The Aging Process

A study conducted by Folkerts et al. investigated how humans adapt or will adapt to heat stress caused by climate change over a long-term interval. They used three commonly used methods over a period of 23 years for older adults (≥65 years), in the Netherlands. The methods used were; minimum mortality temperature (MMT), defined as the mean daily temperature at which the lowest mortality occurs; heat sensitivity, the percentage change in mortality per 1°C above the MMT threshold, or heat attributable fraction, the percentage relative excess mortality above MMT. The key findings suggest that despite differences in the three different methods for determining MMT, the susceptibility of humans to heat decreases over time. This work suggests that future epidemiological research should focus on what factors (e.g., physiological, behavioral, technological, or infrastructural adaptations) influence human adaptation the most. Furthermore, this should lead to the promotion of specific adaptation policies for the general population.

## Physiological Mechanisms and a Novel Heat Acclimation Method

Heat acclimation results in physiological adaptations dependent upon the duration of acclimation, type, mode of exercise, and environmental conditions employed. Research by Oberholzer et al. employed prolonged (5.5 weeks, 5 days.wk^−1^) heat acclimation to determine hematological changes and reported that hemoglobin mass was increased by 3% and this was weakly correlated with plasma volume expansion. The mechanism for the hemoglobin mass increase may therefore be a compensatory response in erythropoiesis, secondary to the plasma volume expansion. However, a study conducted by Kampmann and Bröde that employed prolonged heat acclimation over a minimum of 3 weeks, walking for 3 h per session showed that energy expenditure (Q_10_ effect) and thermal cardiac reactivity were unaltered by heat acclimation status. Overall, oxygen uptake increased by 7% and heart rate by 39–41 bpm, per degree increase in rectal temperature, which was independent of heat acclimation. Therefore, this has implications for the methods and models employed, using Q_10_ effect and thermal cardiac reactivity, for workplace heat stress assessments.

With more sporting' events taking place in hot environments, and global warming increasing temperatures, novel and practical acclimation methods are being investigated and employed. Heathcote et al. investigated the effects of the length of time delay (10 min, 1, 8 h) between running in temperate conditions and the acute thermoregulatory responses to 30 min of hot water immersion (39°C). Findings recommended undertaking hot water immersion within 10 min of completing training to maximize core temperature and heart rate responses and thus acclimation stimulus.

## Adaptation To the Heat For Special Populations Including Males, Females, Military Personnel, and Paralympic Athletes

Heat acclimation is widely accepted as the primary intervention one can adopt to optimize performance and work output in the heat (Racinais et al., 2015). In a meta-analysis, Benjamin et al. examined the magnitude of change in maximal oxygen consumption (V°O_2*max*_), time to exhaustion, time trial, mean power, and peak power tests following heat acclimation. It was shown that acclimation provided the largest performance enhancement in time to exhaustion tests, followed by time trial, mean power, and peak power tests. The authors identified several factors that had affected the results of these performance tests, such as the method of heat acclimation induction, fitness level of participants and heat index.

Research by Mikkelsen et al. examined whether heat acclimation adaptations improved aerobic capacity and performance in cool conditions by comparing two groups of participants training for 5.5 weeks, one in the heat and the other in cool/control conditions. The authors noted that when tested in cool conditions, participants having been heat acclimated did not improve peak power output or aerobic capacity during atest. However, time trial performance in cool conditions improved in the heat acclimation group, albeit to a similar extent than the cool/control group. They concluded that training in the heat is not superior to training in normal conditions for improving aerobic power or time trial performance in cool conditions.

In a more short-term heat acclimation and sprint performance-oriented paper, Garrett et al. focused on the performance responses of female participants when controlling for menstrual cycle. Results indicated an improvement in mean power during maximal sprinting across the 5-day intervention, in line with reductions in core temperature and heart rate, and an increased plasma volume.

Military personnel face unique situations, such as short-notice deployment to hot operational environments that can present medical, occupational, and logistical challenges. In their review, Parsons et al. characterize the physical challenges that military training and deployment present, consider how heat acclimation can augment military performance in hot environments, and identify potential solutions to optimize the risk-performance paradigm.

With a focus on para-triathletes, Stephenson et al. investigated the effectiveness of mixed, active and passive heat acclimation with a controlled heart rate approach at inducing adaptation relative to able-bodied triathletes. The authors reported that para-triathletes displayed partial heat acclimation (i.e., thermoregulatory adaptations) but the extent to which was greater in the able-bodied cohort.

## Heat Therapy For Special Populations

Musculoskeletal injuries disrupt the training, competition and careers of many athletes. Often presented as deconditioning of the cardiovascular, metabolic, and/or muscular systems depending upon the severity of the injury. A review by Ihsan et al. considered an alternative, therapeutic use for heat acclimation that provided evidence for repeated heat exposures embedded into the re-conditioning programs, as part of a *rehabilitation toolbox*, to assist athletes' recovery from injury.

Heat dissipating mechanisms are compromised in individuals with a spinal cord injury, especially in tetraplegia or high-level paraplegia. A review by Zhang and Bishop evaluated the risk of heat injury during training and competition amongst spinal cord injury athletes in order that risk stratification could lead to systematic heat policy to improve knowledge, medical support and protection for these athletes.

A review by Hunt et al. present a compelling perspective on the role of heat therapy and/or heat acclimation that may mediate the upregulation of heat shock proteins (HSP), as an intervention for protecting neurodegeneration in Alzheimer's and Parkinson's disease. The HSP response has been used as a marker of inflammation and heat adaptation. With the pathophysiology of these neurodegenerative diseases linked to loss of protein homeostasis, the authors suggest a heat-induced elevation in HSP from tolerable heat therapy, may improve neuromuscular function, cerebral blood flow, metabolic health, and quality of life. Furthermore, the use heat therapy may lend itself for the treatment of cardiovascular or metabolic diseases.

In summary, as more people travel from cooler to hotter climates, global temperatures increase and more occupations require people to work in hot environments, often at short notice, a better understanding of the individual benefits of heat acclimation is required. The need to understand how heat acclimation can alleviate thermal strain and enhance performance in all humans is essential. Furthermore, it is clear that the “one size fits all” approach may not be appropriate for all populations, as there are often special needs required for heat adaptation. From a sporting perspective, this information is important given the preparations required for the heat and humidity of the 2021 Olympics in Tokyo, Japan (Gerrett et al., 2019). Importantly, the potential benefits of the use of heat therapy for special populations including the treatment of chronic disease remains largely unexplored and needs a great deal more attention.

## Author Contributions

All authors listed have made a substantial, direct and intellectual contribution to the work, and approved it for publication.

## Conflict of Interest

The authors declare that the research was conducted in the absence of any commercial or financial relationships that could be construed as a potential conflict of interest.
